# Revue Homœopathique Francaise

**Published:** 1890-03

**Authors:** 


REVUE HOMŒOPATHIQUE FRANÇAISE. Paris, 11 Rue D'Aumale, Tome I, No. 1.

This is the first number of a new journal devoted to Homœopathy. It will be published and controlled by "La Société Française d'Homœopathie," a new society formed by a fusion of "La Société Homœopathique de France" and "La Société Hahnemannienne Fédérative," and is the outgrowth of the Homœopathic Congress that assembled at Paris, in August, 1889.

The new journal will have forty-eight pages, and be issued *ten* times a year, on the last day of the month.

While containing everything usually to be found in a homœopathic journal, it will be especially devoted to full reports of the meetings of the new society by which it was founded. W. M. J.

